# Occurrence of dMMR/MSI-H tumor during follow-up in Lynch syndrome patients treated with immune checkpoint inhibitors for metastatic digestive cancer between 2015 and 2024: a retrospective analysis of a monocentric prospective cohort study

**DOI:** 10.1016/j.esmoop.2025.105559

**Published:** 2025-09-09

**Authors:** A. Pellat, C. Loisel, J. Metras, J.H. Lefevre, Y. Parc, R. Cohen, T. Samaille, A. Perrier, A. Dardenne, T. André

**Affiliations:** 1 Gastroenterology, Digestive Endoscopy and Digestive Oncology Department, Cochin Hospital, Assistance Publique Hôpitaux de Paris (APHP), Paris, France; 2 Université Paris Cité and Université Sorbonne Paris Nord, Inserm, INRAE, Centre for Research in Epidemiology and Statistics (CRESS), Paris, France; 3 Saint Antoine Hospital, Assistance Publique Hôpitaux de Paris (APHP), Sorbonne Université, Paris, France; 4 Gastroenterology Department, Hôpital Pontchaillou, Rennes 1 University, Rennes, France; 5 Department of Genetics, Pitié-Salpêtrière Hospital, Assistance Publique Hôpitaux de Paris (AP-HP), Sorbonne Université, Paris, France; 6 Unité Mixte de Recherche Scientifique 938, SIRIC CURAMUS, Paris, France; 7 Department of Digestive Surgery, Saint Antoine Hospital, Assistance Publique Hôpitaux de Paris (APHP), Sorbonne Université, Paris, France

**Keywords:** Lynch syndrome, dMMR/MSI-H cancer, gastrointestinal cancer, colorectal polyps, immune checkpoint inhibitors

## Abstract

**Background:**

Immune checkpoint inhibitors (ICIs) represent a paradigm shift and a therapeutic revolution in the management of mismatch repair-deficient/microsatellite instability-high (dMMR/MSI-H) colorectal cancer (CRC), and therefore for patients with Lynch syndrome (LS). The risk of developing metachronous cancers and colorectal polyps in a population of LS patients treated with ICI(s) is not well understood.

**Materials and methods:**

In a single-center cohort study, we retrospectively reviewed 93 LS patients from the prospective ‘ImmunoMSI’ cohort, who were diagnosed with dMMR/MSI-H gastrointestinal cancer and were treated with ICIs for index metastatic gastrointestinal cancer between February 2015 and April 2024. The primary and secondary outcomes were the cumulative risks of metachronous dMMR/MSI-H cancer(s) and *de novo* colorectal preneoplastic polyps, respectively.

**Results:**

The median age at diagnosis was 62 years (range 26-77 years). Most patients carried a germline pathogenic variant in the *MSH2*/*EPCAM* (47%) or *MLH1* (30%) genes. The majority presented CRC as their index cancer (83%), followed by cancers of the small intestine (6%), stomach (4%), and pancreas (4%). During or after treatment with ICI(s), 8 out of 93 patients (8%) developed a total of 9 dMMR/MSI-H cancers, including urothelial (*n* = 4), colorectal (*n* = 3), pancreatic (*n* = 1), and skin cancer (*n* = 1). All patients who developed metachronous cancers had either a complete or partial response to ICI(s) treatment for their index cancer. During a median follow-up of 47.8 months, 44 patients had colonoscopies, and 17 (39%) developed *de novo* preneoplastic polyps.

**Conclusions:**

Our results show that patients with LS treated with ICIs for dMMR/MSI-H metastatic digestive cancers are at risk of developing metachronous dMMR/MSI-H cancers and preneoplastic colorectal polyps. This underlines the importance of dedicated long-term follow-up in this population, even after successful ICI treatment for their first digestive cancer.

## Highlights

• Patients with LS treated with ICIs are at risk of metachronous dMMR/MSI-H cancers (8% of cohort).
• The most frequent metachronous cancers observed were urothelial cancer and CRC.
• Preneoplastic colorectal polyps developed in 39% of patients undergoing colonoscopies during follow-up.
• Long-term surveillance is critical for LS patients post-ICI treatment.

## Introduction

Lynch syndrome (LS) is the most common cause of hereditary colorectal cancer (CRC), accounting for 3% of all CRC.^1^^,^^2^ It results from an inherited mutation in one of the DNA mismatch repair (MMR) system genes (*MSH2/EPCAM*, *MLH1*, *PMS2*, *MSH6*, *MLH1* somatic methylation), leading to the loss of function of the corresponding MMR protein.^3^ Patients with pathogenic variants in *MLH1* and *MSH2* have a higher risk of developing cancer than those with mutations in *MSH6* and *PMS2.*^4^, ^5^, ^6^, ^7^ These mutations lead to microsatellite instability-high (MSI-H) status, significantly increasing the risk of cancers, particularly of the colon and rectum, endometrium, urinary tract, and small intestine. Individualized screening is essential for CRC prevention in LS patients. Current European guidelines recommend high-definition colonoscopy every 2 years starting at age 25 years in patients with an *MSH2/MLH1* mutation and at age 35 years for those with an *MSH6/PMS2* mutation.^8^

LS is associated with a broad spectrum of cancers beyond CRC, including small intestine, gastric, biliopancreatic, and urothelial cancers. Recent studies suggest an expanded spectrum that may include other tumors such as medullary breast cancer^7^ and cutaneous squamous-cell carcinoma.^9^^,^^10^ Muir–Torre syndrome, a phenotypic variant of LS, is characterized by the association of visceral cancer of the LS spectrum with sebaceous skin tumors or keratoacanthomas.^11^

In recent years, immune checkpoint inhibitors (ICIs) have transformed the treatment and prognosis of metastatic MMR-deficient (dMMR)/MSI-H cancers,^12^, ^13^, ^14^, ^15^ which frequently occur in the context of LS.^14^ While the risk of metachronous dMMR/MSI-H cancers in LS patients after a first cancer is well established,^16^^,^^17^ the effect of ICIs on preventing new metachronous dMMR/MSI-H cancers or preneoplastic lesions such as dysplastic or serrated colorectal polyps remains unknown due to limited and poor data.^18^

Our study aimed to evaluate the risk of developing metachronous cancers and preneoplastic colorectal polyps in LS carriers treated with ICIs for an index metastatic digestive cancer.

## Materials and methods

### Study population

We collected data on patients from the prospective ‘ImmunoMSI’ database that included patients who had dMMR/MSI-H metastatic digestive cancer treated with ICI(s) between February 2015 and April 2024 at Saint-Antoine Hospital (Paris, France). The database was approved by the ethics committee (CER-2024-AMARA-ImmunoMSI—MS1.0-00267). Non-objection to the use of routine care data and database information was obtained for all study patients, unless deceased. This cohort was approved by the ethics committee (N°2020—CER 2020-6).

In our study cohort we included only patients who were >18 years of age, were treated with ICIs (anti-programmed cell death protein 1 or anti-programmed death-ligand 1 ± anti-cytotoxic T-lymphocyte-associated protein 4), and who had received at least one injection of treatment for a metastatic dMMR/MSI-H digestive cancer (index cancer). The dMMR/MSI-H status was determined through immunohistochemistry and/or multiplex PCR. Patients were selected using the retrospective ImmunoMSI database after confirmation of a germline mutation consistent with a diagnosis of LS. We excluded patients who were previously treated with ICIs, carriers of a ‘Lynch-like syndrome’, or had acquired *MLH1* hypermethylation. To be included in the colonoscopic follow-up analysis, patients had to have at least two successive colonoscopies, both carried out under ICI treatment or with a baseline colonoscopy carried out within 6 months preceding the introduction of treatment with ICI. Patients with a history of a total proctocolectomy or fewer than two colonoscopies were excluded from this analysis.

### Outcome measures

The primary outcome was the cumulative risk of metachronous dMMR/MSI-H cancers following initiation of ICI therapy. Our secondary outcome was the cumulative risk of developing colorectal preneoplastic polyps, including adenomatous polyps, serrated lesions, and non-rectosigmoid hyperplastic polyps of >1 cm. Histological discrimination between hyperplastic polyps and sessile serrated lesions can be difficult, and because hyperplastic polyps are more common in the left-sided colon, right-sided serrated lesions are often misclassified as hyperplastic polyps.

### Data collection

We collected demographic data, personal and family history of cancer, anatomopathological and molecular characteristics of the index cancer treated with ICIs, constitutional pathogenic variants, personal history of polyps, data on pre- and post-ICI colonoscopy results, information on the index cancer, prior treatments, responses to ICI treatment, and any information about any cancers or polyps arising during treatment or after ICI treatment. These data were sourced from the ImmunoMSI database and patient medical records.

Pathogenic variants were classified into four categories by an expert in biology and medical genetics at the Pitié Salpêtrière Hospital (APHP): missense/indel phase, splicing, large rearrangement, and truncating mutation (nonsense mutation and frameshift). Variant interpretation was carried out according to the latest guidelines from the American College of Medical Genetics and Genomics.^19^

### Statistical analysis and definitions

Categorical variables were described by their number and frequency, while continuous variables by their mean and standard deviation or median and interquartile range (IQR). The baseline colonoscopy was defined as the first colonoscopy carried out under or after ICI treatment, or <6 months before the introduction of ICI therapy. Only preneoplastic polyps (adenomatous polyps, serrated lesions, and non-rectosigmoid hyperplastic polyps of >1 cm) were included in the analysis.^20^^,^^21^ Polyps visualized and resected during the baseline colonoscopy were not included in the final polyp count, to avoid bias from including polyps that existed before ICI treatment. On the contrary, if a polyp was not resected, it was included in the final polyp count and not counted twice.

Survival analyses for each endpoint were carried out using the Kaplan–Meier method.

For all analyses, statistical significance was set at *P* < 0.05. Data analysis was carried out using Rstudio software version 1.2.5033 (Posit PBC, Boston, MA), and the study followed the ESMO-GROW reporting guidelines.^22^

## Results

**Figure 1:**
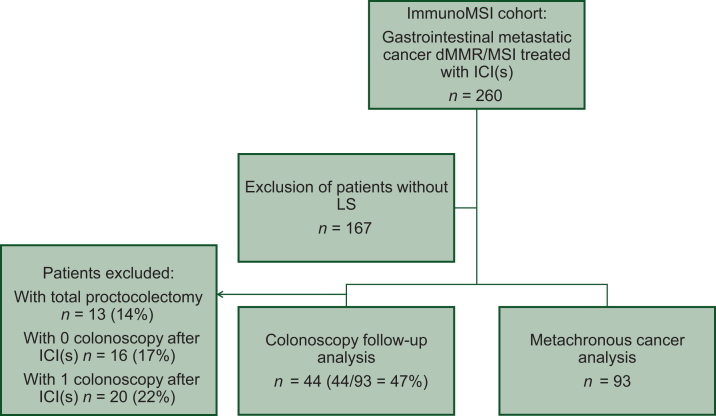
**Flow chart.** dMMR, mismatch repair-deficient; ICI(s), immune checkpoint inhibitor(s); LS, Lynch syndrome; MSI, microsatellite instability.

Among the 206 patients included in the ImmunoMSI cohort, 93 patients with constitutionally confirmed LS and a known germline mutation were included in this analysis (Figure 1). These 93 patients were assessed for the occurrence of metachronous dMMR/MSI-H cancer, while 44 (47%) of them were analyzed for colonoscopic follow-up after excluding those who had undergone total proctocolectomy or had zero or only one colonoscopy.

The median follow-up time of the population from the first day of ICI treatment to the date of last news or death was 47.8 months (IQR 25-75: 22.2-69.2 months).

Characteristics of the study population are summarized in Table 1. The median age at diagnosis of index cancer was 62 years (range 26-77 years). The majority of index cancers were CRC (83%), followed by cancers of the small intestine (6%), stomach (4%), pancreas (4%), and bile ducts (1%). The most frequent pathogenic variant was located on *MSH2* (47%), with truncating variants being the most frequently identified pathogenic mutations (34%).

**Table 1:** Characteristics of the study cohort (*n* = 93)

Characteristics	Overall cohort, *n* (%)
Age (years)	
Median (min-max)	62 (26-77)
Sex	
Male	62 (67)
Performance status	
≤1	89 (95)
Localization of the primary tumor	
Colorectal	78 (83)^a^
Right	52
Left	23
Rectum	12
Pancreas	4 (4)
Stomach	4 (4)
Small intestine	6 (6)
Bile ducts	1 (1)
Gene	
*MSH2/EPCAM*	44 (2 *EPCAM*) (47)
*MLH1*	28 (30)
*MSH6*	14 (15)
*PMS2*	4 (4)
*MLH1* promoter methylation	3 (4)
Type of mutation	
Missense mutation/indel	19 (21)
Splicing	20 (22)
Large rearrangement	14 (15)
Truncating mutation	30 (34)
Missing data	7 (8)
Personal history of cancer (LS spectrum) before ICI-treated metastatic cancer	39^b^ (42)
Surgery before ICI	
Total proctocolectomy	12 (13)
Subtotal colectomy	5 (5)
Partial colectomy/proctectomy	66 (71)
No surgery	10 (11)
Liver metastases	46 (49)
Peritoneal carcinosis	51 (55)
MMR status	
pMMR	2^c^ (2)
dMMR	91 (98)
MSI status	
MSI-H^d^	71 (76)
Missing data	22 (24)
Chemotherapy before ICI(s) for the index cancer	69 (74)
Previous progression to ICI	14 (15)

CRC, colorectal cancer; dMMR, MMR-deficient; ICI, immune checkpoint inhibitor; LS, Lynch syndrome; MMR, mismatch repair; MSI, microsatellite instability; MSI-H, MSI-high; PCR, polymerase chain reaction; pMMR, MMR-proficient.

a Among 78 patients with at least 1 colorectal primary tumor, 6 patients had multiple tumors (2/3 locations), resulting in a total of 87 colorectal tumors.

b 33 out of 39 patients had a history of CRC.

c Among the 93 cancers analyzed, 2 were pMMR but exhibited MSI detected via pentaplex PCR analysis. Both tumors were adenocarcinomas of the right colon, occurring in the context of known LS.

d All cancers tested by PCR showed an MSI-H phenotype, with MSI-H defined as instability of at least two markers in pentaplex PCR molecular analysis.

### Primary outcome: development of metachronous dMMR/MSI-H cancers

In our cohort, 8 out of 93 patients (8.6%) developed metachronous dMMR/MSI-H cancers, with a total of 9 dMMR/MSI-H cancers (1 patient developed 2 metachronous cancers), including urothelial (*n* = 4), colorectal (*n* = 3), biliopancreatic (*n* = 1), and skin (*n* = 1) cancers. All metachronous cancers were diagnosed at a localized stage. The median time to development of metachronous dMMR/MSI-H cancer from the start of exposure to ICI was 27.6 months (IQR 16.1-37.7 months). Additionally, 3 MMR-proficient (pMMR) cancers were recorded, including 2 breast cancers and 1 lung adenocarcinoma, bringing the total number of new cancers in our cohort to 12.

In the population of 93 patients treated with ICIs, the annual incidence rate of dMMR/MSI-H cancers was 2.5% over a cumulative period of 363 patient-years. The annual incidence rate of all cancers combined (dMMR and pMMR) was 3.3% over the same period.

The characteristics of the patients with dMMR/MSI-H metastatic digestive cancers are summarized in Table 2. The median duration of ICI treatment for patients who developed metachronous cancer was 14.8 months (ranging from 8.2 weeks to 39.4 months).

**Table 2:** Description of patients with dMMR/MSI metachronous cancers (eight patients with a total of nine cancers)

Gene mutation	Index cancer	Metachronous cancer	MMR status of metachronous cancer	ICI(s) status when occurrence	Best response with ICI(s) index cancer	Secondary progression of index cancer
*MSH2*	CRC	Urothelial carcinoma	dMMR	Post	PR	No
*MLH1*	CRC	Synchronous CRC: transverse colon and the rectal	dMMR × 2	Post	PR	Yes
*MSH6*	CRC	Urothelial carcinoma	dMMR	During	CR	No
*MSH2*	CRC	Urothelial carcinoma	dMMR	Post	PR	No
*MSH2*	Pancreatic	SSCC	dMMR	Post	CR	No
*MSH2*	Pancreatic	Pelvic recurrence of a rectal cancer	dMMR	During	PR	No
*MSH2*	CRC	Pancreatic	dMMR	Post	CR	No
*MSH2*	CRC	Urothelial carcinoma	dMMR	Post	PR	No

CR, complete response; CRC, colorectal cancer; dMMR, MMR-deficient; MMR, mismatch repair; PR, partial response; SSCC, skin squamous-cell carcinoma.

Two patients developed multiple cancers, including one who developed two metachronous dMMR/MSI-H CRCs and another who developed both a dMMR/MSI-H urothelial carcinoma and a pMMR lung adenocarcinoma, both occurring after the end of ICI treatment. Only two patients experienced the occurrence of metachronous cancers during ICI treatment and not after interruption of ICIs.

All patients who developed metachronous cancers had index cancers that had responded to ICI treatment (partial response or stabilization). Only one patient showed progression secondary to ICI treatment 3 years after the end of treatment and 1 year after the diagnosis of metachronous cancer.

In all cases, metachronous cancers were surgically treated. Six of the eight patients had a prior history of cancer before index cancer, and among those with CRC, the primary tumor was not present at the time of metachronous cancer development. Of the patients who developed a metachronous cancer, 75% (six out of eight) carried an MSH2/EPCAM mutation.

The cumulative incidences of metachronous cancers were 1.2% at 12 months [95% confidence interval (CI) 0% to 3.7%], 3.9% at 24 months (95% CI 0% to 8.2%), and 7% at 36 months (95% CI 0.1% to 12.8%; Supplementary Figure S1, available at https://doi.org/10.1016/j.esmoop.2025.105559).

### Colonoscopy follow-up and development of preneoplastic polyps

Sixty-four patients (69%) in the cohort underwent at least one colonoscopy during ICI treatment or within the 6 months preceding the initiation of ICI therapy. Of these, 44 were included in the analysis of colonoscopic follow-up, as they had at least two successive colonoscopies (including the baseline colonoscopy). These patients were monitored with regular colonoscopies at intervals of 1-2 years, with a median of 2 colonoscopies per patient (range 2-4). During the colonoscopy follow-up period, 1 patient (described in Table 2) presented with synchronous CRC (transverse and rectum), and 17 out of 44 patients (38.6%) developed preneoplastic colorectal polyps. Of these 17 patients, 16 had a history of partial colectomy. The cumulative risk of developing a preneoplastic polyp in the whole cohort was 0% at 12 months, 13.9% (95% CI 2.9% to 23.6%) at 2 years, and 22.9% (95% CI 9.7% to 35.6%) at 3 years following the baseline colonoscopy. A total of 41 polyps were encountered in these 17 patients (Table 3). The median time to polyp development following the baseline colonoscopy was 31.0 months (IQR 25-75: 24-30 months).

**Table 3:** Characteristics of patients with preneoplastic polyps (*n* = 17)

Gene mutation	Index cancer	History of colectomy before ICI	Previous polyps before ICI	Number of polyps during follow-up	Best response with ICI	ICI status
*MLH1*	CRC	Partial	No	1	CR	During
*MSH2*	CRC	Partial	Yes	1	PR	Post
*MSH2*	CRC	Partial	No	1	PR	Post
*MSH2*	CRC	Partial	Yes	8	CR	Post
*MLH1*	CRC	Partial	No	3	CR	Post
*MLH1*	CRC	Partial	Yes	1	PR	Post
*MSH2*	CRC	No surgery	Yes	3	CR	During
*MSH6*	CRC	Partial	No	3	PR	During
*MSH6*	CRC	Partial	Yes	1	PR	During
*MSH2*	CRC	Partial	Yes	5	PR	Post
*MSH2*	CRC	Partial	No	1	SD	Post
*MSH2*	CRC	Partial	No	1	CR	Post
*PMS2*	CRC	Partial	No	3	CR	Post
*MLH1*	CRC	Partial	No	2	PR	Post
*MLH1*	Small intestine	Partial	Yes	2	CR	Post
*MSH2*	Pancreas	Partial	No	3	PR	Post
*MLH1*	CRC	Partial	No	2	CR	Post

CR, complete response; CRC, colorectal cancer; ICI, immune checkpoint inhibitor; PR, partial response; SD, stable disease.

Among the 41 polyps identified, the majority were adenomatous polyps with low-grade dysplasia (56%), followed by sessile serrated polyps without dysplasia (17%), and hyperplastic polyps located in the left or transverse colon (17%). Four patients were found to have adenomas with high-grade dysplasia. Notably, three out of the four patients who developed high-grade dysplasia during follow-up carried a pathogenic variant in *MSH2* (Table 3).

Of the 17 patients who developed preneoplastic polyps, 8 had a variant in *MSH2*, 6 in *MLH1*, 2 in *MSH6*, and 1 in *PMS2*. The majority of these patients (77%) developed polyps after discontinuation of ICI therapy, while 33% developed them during ICI treatment. The median number of polyps per patient was 2 (range 1-8). Additionally, 16 out of 17 patients (94%) who developed preneoplastic polyps had a history of partial colectomy.

The median duration of ICI treatment in the group of patients who developed preneoplastic polyps was 23.8 months (ranging from 6 weeks to 80.7 months). Of the 17 patients, 16 showed a complete or partial response under ICI treatment for index metastatic cancer (Table 3).

## Discussion

In our study, 8 out of 93 patients (8.6%) with LS developed metachronous dMMR/MSI-H cancers despite a response of the index cancer under treatment with ICIs, and 3 developed pMMR/MSS cancers. Among the eight patients who developed metachronous dMMR/MSI-H cancers, 75% carried a pathogenic variant in *MSH2*, suggesting that this subgroup of LS patients has a higher probability of developing metachronous cancer compared with those with other gene mutations.

Previous data have shown that patients with LS who develop a primary non-metastatic CRC have a 32% risk of developing a metachronous cancer after a median follow-up of 53 months, with a majority of metachronous cancers being CRC.^23^ To date, there are limited data on the risk of metachronous cancers in patients with metastatic gastrointestinal cancers due to the shorter survival of patients in historical cohorts.^7^^,^^24^

The mean annual incidence of cancer in our cohort of 93 patients was 3.3%. Similarly, the incidence rate of metachronous cancer in an American cohort of patients not exposed to ICIs was 3.2% per year of observation.^7^ Our results suggest that while ICIs are effective in treating index metastatic dMMR/MSI-H cancers, they do not prevent the development of new cancers, particularly CRC, in LS patients. An American study of 173 LS patients treated with ICIs also observed similar rates of metachronous cancers, with around 12% of new cancers after treatment, mainly cutaneous cancers with a good prognosis. In contrast, our study found that 90% of the new cancers were not cutaneous cancers, with only one dMMR/MSI-H skin cancer observed. This may underestimate the true incidence, possibly due to the lack of specific skin cancer screening guidelines for LS patients in France.^25^ Of note, there are some earlier retrospective studies with contradictory results reporting a decrease in the incidence of secondary dMMR/MSI-H cancers after ICIs treatment, particularly in gastrointestinal cancers.^7^^,^^26^

Colonoscopy follow-up revealed a metachronous CRC in 1 patient, and 17 patients (39%) undergoing colonoscopy developed preneoplastic polyps. The majority were low-grade dysplastic adenomas, while 10% exhibited high-grade dysplastic adenomas. While our findings suggests that ICI(s) do not seem to prevent colorectal polyp development, a Chinese study showed that some adenomas ≥7 mm left in place disappeared after ICIs treatment, suggesting there might be efficacy of this treatment on some polyps.^27^ These results should be interpreted with caution, however, due to variation in adenoma detection rates and colonoscopy quality. Moreover, a study by Yurgelun et al.^28^ found that adenomatous polyps ≥8 mm in LS patients were predominantly dMMR, while nearly 70% of adenomatous polyps ≤5 mm were pMMR, suggesting a potential lack of response to ICIs treatment in the early stage of adenoma development. Furthermore, it has been shown that there may be heterogeneity in dMMR/MSI-H status within the same polyps.^29^ Our results underscore the need for continued colonoscopic surveillance in LS patients who have not undergone total proctocolectomy and are being followed up during or after treatment of metastatic dMMR/MSI-H digestive cancer. A phase II study (NCT03631641) is currently under way to evaluate the preventive use of administration of ICIs to prevent adenomas in patients with LS.^30^

Our study has several strengths. It constitutes a cohort of 93 patients with LS treated with ICIs, with the longest median follow-up reported to date. To minimize classification bias, only patients with confirmed genetic diagnosis of LS were included. In contrast to the American study published in 2023, we specifically selected only metastatic patients given the difference in prognosis in localized dMMR/MSI-H cancers.^31^ We also precisely described the nature of metachronous cancers and their MMR status. Also, patients were recruited from an expert LS center with a multidisciplinary team of experts and organized follow-up. Colonoscopy data were collected according to strict protocols to exclude polyps developed before ICIs treatment, thus avoiding overestimation of the incidence of polyps under treatment.

Several limitations, however, should be noted. Firstly, it is retrospective and single center, which may introduce selection bias. We included only patients with metastatic dMMR/MSI-H digestive cancer treated with ICIs, which could limit the generalizability of our findings. Additionally, there is variability in the number of lines of therapy and treatments received due to the diversity of clinical trials and labeling providing access to ICIs during this period. Another limitation is the inclusion of 14 patients with progressive disease from the start of ICIs treatment, which limited the ability to conduct long-term follow-up on their development of metachronous cancers. In previous cohorts, poor overall survival in patients treated with standard of care without ICIs made it difficult to assess the incidence of metachronous cancers or polyps.^32^

Regarding the analysis of colonoscopic follow-up, the main limitations include the small sample size, the lack of longitudinal data with a median of one colonoscopy per patient, and variability among operators, as each procedure was carried out by different practitioners.^33^

### Conclusion

Despite the demonstrated efficacy of ICIs in treating metastatic dMMR/MSI-H digestive cancers in LS patients, patients seem still at risk of development of metachronous cancers or new colorectal preneoplastic lesions. This underscores the importance of continued surveillance for other primary cancers within the Lynch spectrum, routine colonoscopies, and follow-ups at centers with expertise in LS. Future prospective clinical trials are needed to evaluate the potential preventive role of immunotherapy (ICIs and/or vaccines) in reducing cancer risk in patients with LS.
