# Supplementary material for: Occurrence of dMMR/MSI-H tumor during follow-up in Lynch syndrome patients treated with immune checkpoint inhibitors for metastatic digestive cancer between 2015 and 2024: a retrospective analysis of a monocentric prospective cohort study
Source: ESMO Open. 2025 Sep 9;10(9):105559. doi: 10.1016/j.esmoop.2025.105559 (PMC12455125; doi:10.1016/j.esmoop.2025.105559)
Supplement: Supplementary Figure Legend [file mmc2.docx]

**Supplementary Figure S1. Development of second dMMR/MSI cancers after exposure to ICIs in the whole cohort (N = 93)**
